# A formula based on autonomic test using EZSCAN and anthropometric data for diagnosis of DM in China

**DOI:** 10.1038/s41598-020-61841-2

**Published:** 2020-03-17

**Authors:** Xiaolan Zhao, Alexander Getmanenko, Yalan Zhang, Qinyun Mo, Chunyan Yao

**Affiliations:** 1 Department of Transfusion Medicine, Southwest Hospital, Third Military Medical University (Army Medical University), Chongqing, 400038 China; 2 Center of Health Examination, Southwest Hospital, Third Military Medical University (Army Medical University), Chongqing, 400038 China; 3 Department of Mathematics, Universidad de los Andes Colombia, Bogota, Colombia

**Keywords:** Biological techniques, Endocrinology

## Abstract

Clinical diagnosis of diabetes mellitus (DM) is time-consuming and invasive. This study aimed to investigate the efficacy and accuracy of EZSCAN in detecting impaired glucose tolerance (IGT) and diabetes mellitus (DM) in Chinese population, and explore a diagnosis formula based on an autonomic test using EZSCAN measurement and anthropometric data. Eligible subjects (n = 1547) had the following data collected: those of anthropometric and EZSCAN measurements and biochemical tests including FPG, OGTT, HbA1c, and serum lipid tests. The support vector machine (SVM) algorithm method was used to derive a diagnostic formula. In this study, 452 and 263 subjects were diagnosed with T2DM and IGT, respectively, while 832 had normal glucose tolerance (NGT). The sensitivity rates for the formula were 77.2% for T2DM and 80.4% for IGT. The diagnostic formula was found to correlate strongly with EZSCAN values. The diagnostic formula based on autonomic test and anthropometric data appears to be a convenient and accurate routine screening option in the Chinese population.

## Introduction

Diabetes mellitus (DM) is among the most common metabolic disorders, with more than 400 million adults affected worldwide in 2017^1,2^. In China, the most recent nationwide survey of DM prevalence yielded rates of 9.7% and 10.4% in 2010 and 2013, respectively^3,4^. Despite the high prevalence, type 2 diabetes mellitus (T2DM) can be prevented in high-risk individuals through dietary and lifestyle changes^5,6^. Moreover, the early diagnosis of this condition, which is asymptomatic at early stages^7^, can enable appropriate interventions at an earlier point and thus minimize complications.

Current recommended biochemical tests for IGT and T2DM diagnosis include oral glucose tolerance test (OGTT), fasting plasma glucose (FPG), and glycated hemoglobin (HbA1c) tests. Although FPG testing is recommended as an initial screening option for non-pregnant adults^7^, this test has diagnostic sensitivity rates as low as 40% when used alone^8,9^. The OGTT is time-consuming and may be difficult to perform, while the HbA1c test is expensive and has lower sensitivity^10^.

Small fibers may be injured early in the course of DM and affect sudomotor function^11^, thus, sudomotor function assessment could contribute to the detection of autonomic dysfunction in diabetics^4,5^. EZSCAN (Impeto Medical, Pairs, France) system, which can perform a precise evaluation of sudomotor function, was recently adapted for DM screening. EZSCAN is a new, noninvasive and quick method for the precise evaluation of sweat-gland function through electrochemical skin conductance (ESC) measurement^12^. The underlying pathophysiological mechanism involves lesion induction, in which the small autonomic nerve fibers innervating the eccrine glands respond differently to an electric current stimulus due to hyperglycemia^13–16^. This test requires no preparation and takes only 2–3 min to complete.

Several studies have evaluated the correlation of EZSCAN results with actual glucose levels. However, no study has established a definitive cut-off point for further diagnostic testing. One study reported a sensitivity and specificity of 75% and 100%, respectively, for assessing sudomotor dysfunction using EZSCAN in a French population^11^. Similarly, in an Indian population, EZSCAN device yielded sensitivity rates of 75% and 70% for the detection of DM and IGT using a cut-off point of 50%^17^. Different cut-off points for the EZSCAN system were also determined in the Chinese population. Yang *et al*. reported that an EZSCAN value>30% indicated an increased risk of prediabetes and diabetes^18^. Chen *et al*. reported optimal cut-off points of 37% and 50% for the detection of IGT and DM, respectively, although the sensitivity (53%) and specificity rates (59%) in their case were relatively low^10^. Sheng used an EZSCAN index of 40 as the threshold value and reported sensitivity and specificity rates of 85% and 64%, respectively, for the diagnosis of DM^19^. Above studies suggest that in comparison with the French and Indian populations, a lower EZSCAN threshold would be adequate for the diagnosis of IGT and DM in Chinese individuals at reasonable levels of sensitivity and specificity.

The EZSCAN is a device developed primarily for the detection of diabetes and diabetic complications based on sudomotor function. However, only by assessing of the sudomotor function, EZSCAN could be relatively less sensitive and less accurate. Dyck *et al*. suggested that a combination of age at onset of diabetes, HbA1c level, and duration of diabetes predicted impaired glucose tolerance status better than single components of chronic glycemic exposure (the degree or duration of hyperglycemia)^20^. Similarly, Sun *et al*. pointed out that EZSCAN is more suitable for screening purpose and not for diagnosis; they recommend that other factors, such as blood pressure, lipid profiles, and body mass index (BMI) should be considered for a better evaluation of the metabolic syndrome^15^. Based on the above studies, our study aimed to determine a convenient and accurate screening method for IGT and T2DM based on an autonomic test using EZSCAN measurements and anthropometric data from Chinese subjects. The association between autonomic function and impaired glucose tolerance status was explored, and the accuracy of a diagnostic formula was researched.

## Results

### Anthropometric data and EZSCAN test

Of the 1547 enrolled subjects, 452 were diagnosed with T2DM, 263 with IGT, and 832 with NGT. The demographic, clinical, and laboratory characteristics of the participants are presented in Table 1. When compared with the NGT group, subjects in the IGT and T2DM groups were generally older and had a larger waist circumference and higher BMI, SBP, TG, FPG, 2h-OGTT and HbA1c levels. As shown in Table 2, an increasing EZSCAN risk level was associated with increase in age, BMI, waist circumference, SBP, TG, TC, and FPG and a decrease in HDL-C. The EZSCAN scores were significantly higher in the IGT (47 ± 11%) and T2DM groups (58 ± 11%) than in the NGT group (34 ± 13%). Furthermore, the EZSCAN values correlated positively with the 2h-OGTT and FPG levels (r = 0.282 and r = 0.203, respectively, *p* < 0.001). Although the correlations were moderate, they were statistically significant and suggested that impaired glucose tolerance contribute to autonomic dysfunction.

**Table 1:** Demographic data of the subjects defined by OGTT.

Variable (mean ± SD)	NGT (n = 832)	prediabetes (n = 263)	T2DM (n = 452)	*P*
Age (y)	45.6 ± 3.1	47.3 ± 2.7	51.2 ± 2.4	<0.05
Waist circumferences (cm)	78.6 ± 7.4	82.1 ± 7.8	85.3 ± 8.2	<0.01
BMI (kg/m^2^)	23.1 ± 1.9	24.2 ± 2.3	25.6 ± 2.8	<0.01
SBP (mm Hg)	122.6 ± 8.8	129.2 ± 6.7	136.7 ± 10.1	<0.01
DBP (mm Hg)	78.2 ± 11.3	81.6 ± 10.5	82.2 ± 11.2	0.819
FPG (mmol/L)	4.6 ± 0.4	6.3 ± 0.2	7.4 ± 0.3	<0.01
2h-OGTT (mmol/L)	6.1 ± 0.4	9.7 ± 0.5	11.2 ± 0.1	<0.01
HbA1c (%)	5.8 ± 0.1	6.3 ± 0.2	6.9 ± 0.4	<0.05
TC (mmol/L)	4.7 ± 0.5	5.1 ± 0.6	5.5 ± 0.8	0.693
TG (mmol/L)	1.1 ± 0.3	1.3 ± 0.4	1.6 ± 0.4	<0.05
LDL-C (mmol/L)	3.1 ± 0.6	3.6 ± 0.6	4.1 ± 0.6	0.553
HDL-C (mmol/L)	1.8 ± 0.6	1.6 ± 0.6	1.4 ± 0.4	0.167

BMI = body mass index; SBP = systolic blood pressure; DBP = diastolic blood pressure; FPG = fasting plasma glucose; OGTT = oral glucose tolerance test; HbA1c = glycated hemoglobin; TC = total cholesterol; TG = triglycerides; LDL-C = low-density lipoprotein cholesterol; HDL-C = high-density lipoprotein cholesterol; SD = standard deviation.

**Table 2:** Demographic data of the subjects according to EZSCAN value.

Variable (mean ± SD)	EZSCN (0–35)	EZSCN (36–70)	EZSCN (71–100)	*P*
Age (y)	44.2 ± 2.6	45.4 ± 3.5	54.6 ± 3.4	<0.05
Waist circumferences (cm)	77.5 ± 7.4	84.2 ± 7.8	88.7 ± 8.6	<0.01
BMI (kg/m^2^)	22.8 ± 1.7	24.5 ± 2.5	26.5 ± 2.8	<0.01
SBP (mm Hg)	121.1 ± 9.1	130.2 ± 8.9	138.7 ± 10.1	<0.01
DBP (mm Hg)	84.3 ± 11.3	88.6 ± 9.5	90.2 ± 10.2	0.613
FPG (mmol/L)	4.4	6.4	7.6	<0.01
2h-OGTT (mmol/L)	6.1	8.9	11.8	0.821
HbA1c (%)	5.4	7.8	9.1	0.734
TC (mmol/L)	4.1 ± 0.7	5.2 ± 0.9	6.3 ± 0.9	<0.05
TG (mmol/L)	1.1 ± 0.3	1.5 ± 0.4	1.9 ± 0.4	<0.05
LDL-C (mmol/L)	2.8 ± 0.6	3.9 ± 0.6	4.5 ± 0.7	0.615
HDL-C (mmol/L)	2.3 ± 0.6	1.6 ± 0.5	1.1 ± 0.4	<0.05

BMI = body mass index; SBP = systolic blood pressure; DBP = diastolic blood pressure; FPG = fasting plasma glucose; OGTT = oral glucose tolerance test; HbA1c = glycated hemoglobin; TC = total cholesterol; TG = triglycerides; LDL-C = low-density lipoprotein cholesterol; HDL-C = high-density lipoprotein cholesterol; SD = standard deviation.

### Association of formula value with prediabetes and T2DM

**Figure 1:**
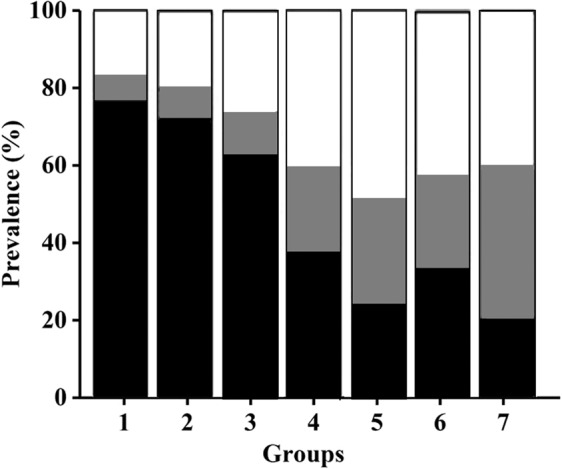
Prevalence of NGT (black part), IGT (grey part), and T2DM (white part) across formula values. Each column represents subjects from a particular group (according to the formula value), while the grey and white scales represent the proportions of IGT and T2DM diagnoses, respectively.

The following seven groups according to formula value were determined: group 1, formula value < −1; group 2, −1 to <−0.5; group 3, −0.5 to <0; group 4, 0 to <0.5; group 5, 0.5 to <1; group 6, 1 to <1.5; and group 7, >1.5. The respective prevalence of T2DM in each group was 20%, 21%, 30%, 37%, 40%, 43%, and 44%, compared to the overall prevalence of T2DM of 29% (Fig. 1). This change of T2DM prevalence was statistically significant (Pearson’s chi-square test, *p* < 0.001), which demonstrated that formula value was closely related to prevalence of T2DM. The odds ratios (ORs) for the seven groups were calculated, the risks of having T2DM increased progressively across the group 1 to group 7. The risk of having T2DM significantly increased in group 5, group 6 and group 7 compared with the group with formula value of 0 (OR of 3.34, 95% CI 2.98–3.83; OR of 2.21, 95% CI 1.41–3.42; and OR of 3.20, 95% CI 2.12–5.40, respectively; *P* < 0.001).

### Accuracy of diagnosis formula

When we applied the diagnostic formula presented in the Materials and Methods section, we obtained correct predictions for 1194 of 1547 subjects that yielded a diagnostic accuracy of approximately 77%. The occurrences of various risk factors and the decision formula used for T2DM diagnosis are shown in Table 3. No single risk factor (age > 45 years, DBP > 80 mmHg, EZSC data> 35) got a diagnostic accuracy better than that of the diagnostic formula (*p* < 0.05).

**Table 3:** Occurrence of various risk factors (age, DBP, EZSCAN) and decision formula in the T2DM diagnosis.

	NGT	prediabetes	T2DM
Age ≤45 y	444 (53.4%)	79 (30.0%)	148 (32.7%)
Age> 45 y	388 (46.6%)	184 (70.0%)	304 (67.3%)
DBP ≤ 80 mmHg	541 (65.0%)	87 (33.1%)	195 (43.1%)
DBP > 80 mmHg	291 (35.0%)	176 (66.9%)	257 (56.9%)
EZSCAN ≤ 35	508 (61.1%)	95 (36.1%)	200 (44.2%)
EZSCAN > 35	324 (38.9%)	168 (63.9%)	252 (55.8%)
Formula <0	675 (81.1%)	135 (51.3%)	271 (60.1%)
Formula> 0	157 (18.9%)	128 (48.7%)	181 (39.9%)

NGT = normal glucose tolerance, IGT = impaired glucose tolerance, T2DM = type 2 diabetes mellitus, DBP = diastolic blood pressure.

**Figure 2:**
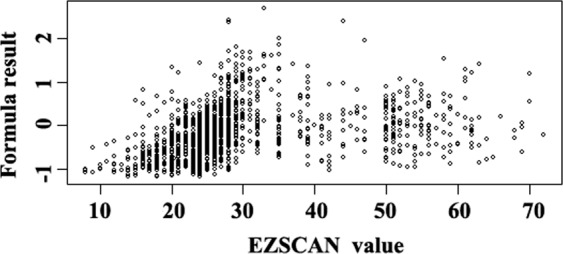
Scatter plots of the relationship of the EZSCAN value with the formula result.

Of the 832 subjects with NGT, 699 (84%) had EZSCAN values of <35. The scatter plot in Fig. 2 demonstrates a good correlation of the formula result with a low EZSCAN value (<35). Therefore, the formula result exhibited a significant correlation with the EZSCAN value (*p*: order of 10^−16^).

## Discussion

There is clear evidence to suggest that sudomotor dysfunction due to small-fibres injury can develop early in diabetes and be detected in diabetic patients during standard clinical evaluation or even earlier in patients with IGT^11^. Given its rapid, noninvasive, and reproducible nature, the EZSCAN device has rapidly attracted attention and has been used to detect sudomotor dysfunction as an early screening method for diabetes in several countries^21–23^. Despite the above advantages, EZSCAN has been limited by a relatively weak detection sensitivity and accuracy^15^. Therefore, we included both EZSCAN and anthropometric risk factors in our exploration of a formula for further screening test, considering the potential mediating effects of factors, such as aging, obesity, smoking, physical activity, and hypertension on the relationship between EZSCAN and T2DM diagnosis. We demonstrated clear associations of the EZSCAN value with some conventional risk factors, including older age and higher SBP. The SVM algorithm method was used to derive decision formula; thus, this formula cannot be significantly improved by any other mathematical method. Our analysis showed that EZSCAN does not perform much better than the age and DBP information together. Combined with previous study, EZSCAN appears to be just a screening tool for high-risk individuals of IGT and DM, and not suitable for diagnosis. EZSCAN and formula values are perfectly correlated (in low value); thus, we concluded that the formula may be a suitable screening tool. Moreover, the diagnostic formula is completely automated, and the results are immediately displayed. In this regard, the diagnostic formula could be suitable for large-scale screening studies. It should be noted that the false-positive rate of the formula method is about 16%; however, keeping in mind the cost of EZSCAN, it would not make an additional financial burden (except for labor costs).

Given the extreme importance of an early assessment of IGT and T2DM risk, any screening tool with the potential clinical ability to diagnose T2DM would be significant in the context of diabetes prevention. The applications of current biochemical tests used to diagnose T2DM in large-scale epidemiological studies and regular follow-ups are limited with respect to the need for professional expertise, expensive instruments, and time-consuming protocols. In China, T2DM is currently diagnosed by the laboratory evaluation of FPG, OGTT, and HbA1c levels, which have respective approximate costs of $1.5, $4.5, and $10. Recently, a point-of-care HbA1c testing, which uses a finger-prick blood sample and provides rapid results within a few minutes, has been applied as a new analyzer for HbA1c determination in some countries^24,25^, however, it is not a regular clinical practice in China.

To our knowledge, this was the largest population-based study to explore the association of EZSCAN with T2DM diagnosis. Additionally, this was the first study to derive a diagnostic formula for T2DM independently of biochemical test data. Additional strengths of this study include its large-scale, population-based design and the inclusion of participants spanning a wide age range (18–75 years), as well as the comparison of EZSCAN results with measured FPG, OGTT, and HbA1c values. However, our study also has several limitations of note. First, all the presented findings were derived from a cross-sectional investigation. Second, each subject underwent only one EZSCAN analysis, and therefore the intra-subject reproducibility could not be examined. In the future, the ability to replicate the current results in distinct populations (especially in long-term studies) would be needed to confirm the usefulness of our formula for the diagnosis of T2DM.

Over the last 30 years, numerous researchers have attempted to develop non-invasive methods of glucose measurement for prediabetes risk assessment. Our findings suggest that our novel diagnostic formula based on an autonomic test using EZSCAN and anthropometric data could be applied to the screening of prediabetes and T2DM in high-risk population, independently of biochemical tests. Nevertheless, the technical improvements on the existing diagnosis method, especially in a convenient and low-cost point-of-care HbA1c testing, are always expected. Based on this, the development of a rapid, automatic, and non-invasive method into a form of novel screening equipment may be of significant interest in the future.

## Methods

### Subjects

This study was conducted between April and October 2015. All subjects had visited Southwest Hospital in Chongqing, China for a routine health check-up during this period. The inclusion criteria were an age of 18–75 years and no history of diagnosis with IGT or DM according to the 2006 World Health Organization (WHO) diagnostic criteria. The following exclusion criteria were also applied: 1) use of drugs that affect blood glucose levels (including corticosteroids, tricyclic antidepressants, diuretics, epinephrine, estrogens, lithium, phenytoin, and salicylates) and/or the sympathetic nervous system (including beta-blockers); 2) history of arm or leg amputation; 3) history of implantation with an electrical device (e.g., pacemaker, defibrillator); 4) known sensitivity to nickel or any other standard electrode; and/or 5) a history of epilepsy or seizures.

A total of 1,547 individuals (52% men) were invited to undergo EZSCAN examinations, anthropometric measurements, and biochemical testing.

### Ethical approval

Informed consent was obtained from all participants and the study protocol was approved by the Medical Ethics Committee of the Southwest Hospital. All subjects gave written informed consent. All the experiments were performed in accordance with the relevant guidelines and regulations.

This article does not contain any studies with animals performed by any of the authors.

### Anthropometric and laboratory measurements

Anthropometric measurements were obtained by trained and certified clinical staff using standard operating protocols and automated electronic devices. Three consecutive blood pressure measurements were obtained at 1-min intervals from all subjects. These measurements were obtained while the subject was in sitting position after 5 min of rest and by the same observer using an automated electronic device (HEM-6200; OMRON Healthcare Ltd., Kyoto, Japan). Height and weight were recorded to the nearest 0.1 cm and 0.1 kg, respectively, while the subject wore light indoor clothing without shoes. Waist circumference was measured at the umbilicus level with the subject in standing position. Blood samples for FPG, HbA1c, and lipid profile analyses were collected after an overnight fast. A standard OGTT was then performed according to WHO recommendations^26^. Plasma glucose was measured using the glucose oxidase method and an autoanalyzer (Modular P800, Roche, Basel, Switzerland). The HbA1c level was determined using high-performance liquid chromatography (BIO-RAD, Hercules, CA, USA). Other biochemical tests were performed using chemiluminescence methods (Modular E170, Roche, Basel, Switzerland). All tests were conducted by researchers blinded to the clinical data.

### Assessment of glucose tolerance status

According to the 2006 WHO diagnostic criteria, diabetes was defined as a FPG level ≥ 7.0 mmol/L or postprandial plasma glucose (PPG) level ≥ 11.1 mmol/L. IGT was defined as a FPG level of 6.1–6.9 mmol/L and/or PPG level of 7.8–11.0 mmol/L. Normal glucose tolerance (NGT) was defined as a FPG level <6.1 mmol/L and PPG level <7.8 mmol/L. Subjects who tested positive for DM by the OGTT were referred to physicians.

### EZSCAN test

All subjects underwent EZSCAN (EZS 01002085441) testing. EZSCAN values (range: 0–100) were derived from the ESC measurements.

### Statistical analysis

Statistical analysis was performed using the SPSS software package, version 16.0 (SPSS, Chicago, IL, USA). The data are presented as means ± standard deviations (SDs), medians, or percentages. For comparisons, Student’s *t*-test and the Mann–Whitney test were applied to continuous variables, while the chi-squared test was applied to categorical variables. Pearson’s correlation analysis and multivariate linear regression models were performed to evaluate the associations of the EZSCAN values with BMI, waist circumference, systolic blood pressure (SBP), diastolic blood pressure (DBP), FPG, 2h-OGTT, triglycerides (TG), total cholesterol (TC), high-density lipoprotein cholesterol (HDL-C), low-density lipoprotein cholesterol (LDL-C), and FPG.

The first stage of data analysis involved feature selection, during which we sought the combination of a limited number of the above parameters (two or three) with the EZSCAN result that would be most informative for the diagnosis of IGT and T2DM. We excluded biochemical test data from this stage because these tests tend to be invasive, costly, and time-consuming, thus contradicting the aim of identifying an alternative method. The following analysis therefore included only anthropometric data.

We selected all possible pairs of the 15 variables with the EZSCAN value. For each pair of variables, we obtained a set of points in three-dimensional space and classified each according to the known diagnosis (0 = NGT, 1 = IGT, and 2 = T2DM). We performed a nearest-neighbor analysis of this set of points to determine whether any diagnostic information could be extracted from the values of the three variables under consideration.

**Figure 3:**
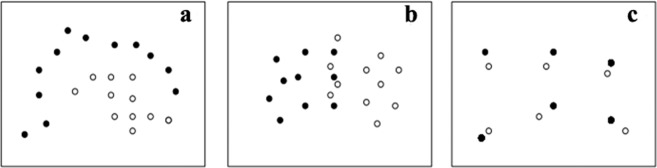
Scatter plots of a given data set which used to predict the labels of new observed points. (**a**) well-separated data set; (**b**) somewhat mixed data set; (**c**) completely mixed data set.

The nearest-neighbor analysis is a well-known mathematical technique. Informally, suppose a huge data set ${x}_{n}$, n = 1,…,N, of points in *k* dimensions (*k* = 3 in our case), each of which is classified as 0, 1, or 2. Further, suppose that we observe a new point, *y*, and would like to predict its label based on previous data. One reasonable approach would be to determine the ${x}_{n}$ closest to *y* in *k*-dimensional space and predict that the two points would have the same label. The efficacy of this approach depends on whether the points with different labels are well separated (Fig. 3a) in the data set ${x}_{n}$, somewhat mixed (Fig. 3b), or completely mixed (Fig. 3c). For the data set shown in Fig. 3a, previous data could be used to predict the labels of new observed points, whereas this was possible with limited success in Fig. 3b and not possible in Fig. 3c, regardless of the statistical method being used. Therefore, this method provides an upper bound for the performance of any other prediction method. The estimation of this performance resembles cross-validation. For each n in the range 1,…, N, we consider the data set of N-1 points, ${x}_{1,\mathrm{..}.},{x}_{n-1},{x}_{n+1},\ldots ,{x}_{N}$, and use this to classify ${x}_{n}$. We then count the number of points, ${x}_{n}$, for which the prediction is correct or incorrect. The percentage of correct predictions thus provides an estimate of the maximal possible percentage of correct predictions based on a given data set.

The purpose of the nearest-neighbor analysis in this study was twofold. First, we used this technique to estimate the accuracy of any reasonable diagnostic method based on the considered variables and achieve an accuracy rate of 77%. Second, this tool helped us select two variables, age and DBP, as the optimal pair in combination with the EZSCAN value using the following approach. Briefly, as 105 pairs of variables were possible (15 × 14/2 = 105), we automatically performed the procedure described below 105 times. Subsequently, we automatically performed the same procedure for all possible triads of variables (15 × 14 × 13/6) combined with the EZSCAN value. This process required approximately 1 day of computing time and allowed us to conclude that the performance of the best triad was not noticeably better than that of the best pair. We therefore proceeded with the combination of age, DBP, and EZSCAN value.

In the next stage, we used the support vector machine (SVM) algorithm included in the R software package e1071 (R Project for Statistical Computing, Vienna, Austria) to analyze the age (year), DBP (mmHg), and EZSCAN data and used these to derive the diagnostic formula. Because the plot of age and DBP against the groups IGT and NGT did not conform to a linear pattern, we chose the following dimensions for our SVM algorithm: X_1_ = Age, X_2_ = Age^2^, X_3_ = DBP, X_4_ = DBP^2^, X_5_ = Age*DBP, X_6_ = EZSCAN. The SVM algorithm implemented in e1071 yielded the following decision formula:

$${\rm{Decision}}=-\,0.611\,{{\rm{Y}}}_{1}+0.553\,{{\rm{Y}}}_{2}-0.524\,{{\rm{Y}}}_{3}+0.557\,{{\rm{Y}}}_{4}+0.605\,{{\rm{Y}}}_{5}-0.0320\,{{\rm{Y}}}_{6}-0.224$$

where Y_1_,…,Y_6_ are the rescaled variables:

$$\begin{array}{lll}{{\rm{Y}}}_{1}=({{\rm{X}}}_{1}-47.6)/9.3 & {{\rm{Y}}}_{2}=({{\rm{X}}}_{2}-2350)/925 & {{\rm{Y}}}_{3}=({{\rm{X}}}_{3}-80.2)/12.9\\ {{\rm{Y}}}_{4}=({{\rm{X}}}_{4}-6602)/2156 & {{\rm{Y}}}_{5}=({{\rm{X}}}_{5}-3830)/1006 & {{\rm{Y}}}_{6}=({{\rm{X}}}_{6}-28.7)/11.1\end{array}$$

If the formula result is positive, the patient would then be classified as IGT or T2DM. If the formula result is negative, the patient would be classified as NGT.

## Data Availability

The datasets generated during and/or analyzed during the current study are available from the corresponding author on reasonable request.
